# Anesthetic spindles serve as EEG markers of the depth variations in anesthesia induced by multifarious general anesthetics in mouse experiments

**DOI:** 10.3389/fphar.2024.1474923

**Published:** 2024-12-13

**Authors:** Ying You, Hui Liu, Zhanfei Yang, Yuxuan Chen, Fei Yang, Tian Yu, Yu Zhang

**Affiliations:** ^1^ Department of Anesthesiology, Affiliated Hospital of Zunyi Medical University, Zunyi, China; ^2^ Key Laboratory of Anesthesia and Organ Protection (Zunyi Medical University), Ministry of Education, Zunyi Medical University, Zunyi, China

**Keywords:** mice, general anesthesia, depth of anesthesia, EEG, spindle activity

## Abstract

**Background:**

Mice play a crucial role in studying the mechanisms of general anesthesia. However, identifying reliable EEG markers for different depths of anesthesia induced by multifarious agents remains a significant challenge. Spindle activity, typically observed during NREM sleep, reflects synchronized thalamocortical activity and is characterized by a frequency range of 7–15 Hz and a duration of 0.5–3 s. Similar patterns, referred to as “anesthetic spindles,” are also observed in the EEG during general anesthesia. However, the variability of anesthetic spindles across different anesthetic agents and depths is not yet fully understood.

**Method:**

Mice were anesthetized with dexmedetomidine, propofol, ketamine, etomidate, isoflurane, or sevoflurane, and cortical EEG recordings were obtained. EEG signals were bandpass filtered between 0.1 and 60 Hz and analyzed using a custom MATLAB script for spindle detection. Anesthesia depth was assessed based on Guedel’s modified stages of anesthesia and the presence of burst suppression in the EEG.

**Results:**

Compared to sleep spindles, anesthetic spindles induced by the different agents exhibited higher amplitudes and longer durations. Isoflurane- and sevoflurane-induced spindles varied with the depth of anesthesia. Spindles associated with etomidate were prominent during induction and light anesthesia, whereas those induced by sevoflurane and isoflurane were more dominant during deep anesthesia and emergence. Post-anesthesia, spindles persisted but ceased more quickly following inhalational anesthesia.

**Conclusion:**

Anesthesia spindle waves reflect distinct changes in anesthesia depth and persist following emergence, serving as objective EEG markers for assessing both anesthesia depth and the recovery process.

## 1 Introduction

Clarifying the mechanisms underlying the loss of consciousness during general anesthesia is crucial for advancing consciousness research within neuroscience. While studying subjective experiences of consciousness directly in animals is challenging, we approach this indirectly by examining specific EEG markers that indicate anesthesia depth. These markers, such as anesthetic spindles, offer objective evidence of brain state transitions, which, in turn, reflect varying degrees of consciousness suppression across anesthesia stages. Different doses of general anesthetics produce varying degrees of consciousness suppression. Although the bispectral index (BIS), derived from electroencephalography (EEG), is a widely used tool in clinical settings to monitor anesthetic depth in humans (Vuyk et al., 2004), relying on algorithms that analyze human EEG patterns to generate a dimensionless score indicative of consciousness levels. However, the BIS algorithm is specifically calibrated to human EEG data and assumes certain brain activity patterns that are not consistently observed across different animal species. Variability in EEG dynamics across species, particularly in response to anesthesia, makes the BIS less reliable for assessing consciousness in animals. Consequently, using BIS in animal studies could lead to inaccurate assessments of anesthesia depth, making it an unsuitable tool for our purposes. In studies investigating the mechanisms of reversible consciousness loss under general anesthesia, many mouse experiments rely on observing burst suppression events in the EEG as indicators of anesthetic efficacy (Li et al., 2019). However, it is important to note that burst suppression occurs only during deep anesthesia and does not reflect the onset of anesthesia or the light anesthesia phase. In current research on the mechanisms of general anesthesia using mice, the loss and recovery of reflexes are commonly used as behavioral indicators to determine the onset and cessation of anesthesia (Wasilczuk et al., 2018). However, this approach only broadly categorizes the anesthesia state into pre-anesthesia, anesthesia, and post-anesthesia phases, without capturing the nuanced effects of different anesthetic agents or the different stages of anesthesia. Therefore, to achieve more precise temporal insights into the mechanisms of general anesthesia, it is essential to identify an objective EEG marker specifically suitable for mice. The study uses a reflex-based methods for assessing anesthesia depth offers a practical, minimally invasive approach that avoids the need for prior interventions. This approach enables us to capture spontaneous transitions in electrophysiological states under different anesthetics without additional preparatory steps. By utilizing these reflex methods can more directly compare electrophysiological changes across anesthesia states. Moreover, by focusing on precise timing of spindle activity and its correlation with anesthesia-induced neural changes, to analyze how specific oscillatory events occur and interact during distinct states of consciousness. This temporal precision is better able to capture the dynamic transitions in neural activity, which are often lost in studies lacking fine temporal resolution. It reliably reflects the distinct effects of different anesthetics and tracks the transition of consciousness from light to deep anesthesia.

Recent research on the mechanisms of general anesthesia often incorporates methods and theories from sleep studies to explore the central processes unique to anesthesia that distinguish it from sleep (Bao et al., 2023). In humans, sleep spindles are distinctive electroencephalographic events that occur during stage 2 of non-rapid eye movement (NREM) sleep, typically characterized by a frequency range of 7–15 Hz and lasting 0.5–3 s (Fernandez and Luthi, 2020). They are detectable in most cortical regions, with higher densities observed in the frontal, somatosensory, and visual areas (Piantoni et al., 2017). The presence of sleep spindles has also been documented across multi-mammal, including mice (Aguilar et al., 2020; Fernandez et al., 2018; Yuan et al., 2021), dogs (Iotchev et al., 2020), and cats (Iotchev and Kubinyi, 2021), indicating that they are evolutionarily conserved sleep-related EEG events. While the amplitude of sleep spindles in rodents is smaller compared to humans, they are nonetheless detectable (Yuan et al., 2021). In rodents, fast and slow spindles are used to further subdivide NREM sleep into fragile and stable phases, suggesting that spindle occurrence may be related to the functional specialization of the sensory cortex (Fernandez and Luthi, 2020; Fernandez et al., 2018; Khazipov et al., 2004).

During NREM sleep, inhibitory gamma-aminobutyric acid (GABA)-ergic neurons within the thalamic reticular nucleus (TRN) undergo membrane hyperpolarization, leading to burst firing activity, which serves as the source of sleep spindles. The TRN then engages in a loop-like interaction with other thalamic nuclei, synchronizing thalamic activity to the spindle rhythm. This spindle activity is transmitted via the thalamocortical (TC) pathway to the cortex, resulting in widespread spindle wave activity across the cortex (Astori et al., 2011). Spindle wave activity reflects thalamo-cortical and cortico-cortical synchronization, suppressing thalamic information transmission and cortical encoding functions, effectively shielding the brain from external stimuli during sleep. Metrics such as spindle density and amplitude are reliable indicators for assessing sleep depth (Chen et al., 2023a).

During general anesthesia, similar spindle-like wave events have been observed, often referred to as anesthesia alpha oscillations. These anesthesia spindle waves are commonly seen with multifarious anesthetic agents, including propofol (Ferenets et al., 2006), isoflurane (Sitdikova et al., 2014), and ketamine (Tsuda et al., 2007), suggesting that they are universal electroencephalographic events during general anesthesia. Some researchers have proposed using spindle waves as markers of anesthesia depth, which could improve the precision of monitoring the suppression of painful stimuli and levels of unconsciousness (Hagihira, 2015; Schonauer and Pohlchen, 2018). However, the feasibility of using anesthesia spindle waves as EEG indicators to reflect anesthesia depth across different anesthetic agents has not been extensively studied.

In this study, we recorded spindle activity induced by two inhalational anesthetics (isoflurane and sevoflurane), three intravenous anesthetics (ketamine, propofol, and etomidate), and one sedative-hypnotic agent (dexmedetomidine) in mice. We observed changes in spindle activity across different stages of anesthesia and conducted comparative analyses of spindle characteristics induced by the different anesthetic agents.

## 2 Methods

### 2.1 Experimental animals

All experiments were conducted using adult C57 BL/6J mice (8–10 weeks old, weighing 20–25 g), obtained from the Animal Experiment Center of Zunyi Medical University (Guizhou, China, Certificate No. SYXK (Qian) 2021–0004). The mice were housed under controlled conditions with a constant temperature (23°C ± 1°C) and humidity (50% ± 5%), maintained on a 12-h light-dark cycle (7:00 AM–7:00 PM), with *ad libitum* access to food and water. All experimental procedures were approved by the Animal Ethics Committee of Zunyi Medical University. The mice were randomly assigned to seven groups, ensuring equal distribution of male and female subjects.

**FIGURE 1:**
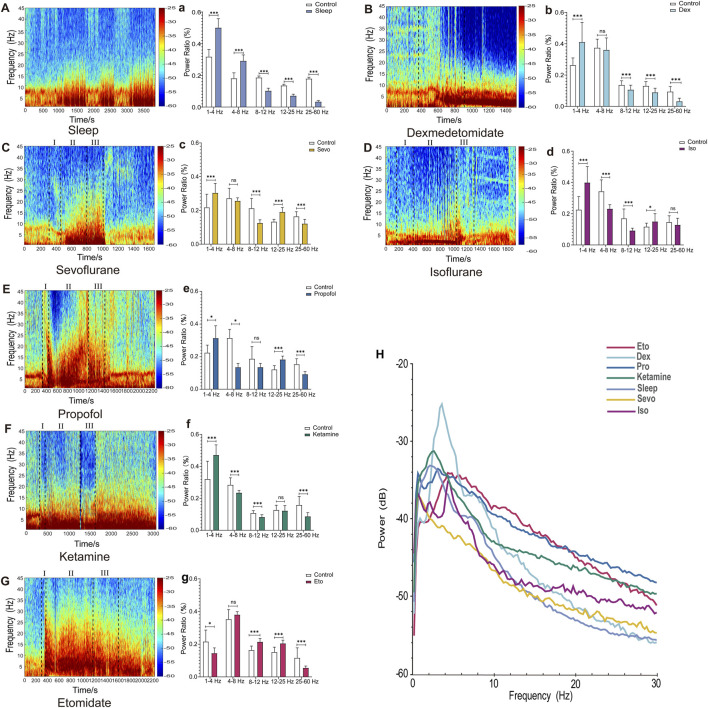
EEG Features of Sleep and drug-induced anesthesia states. **(A–G)** Spectrograms of cortical EEG in mice, with drug administration times marked by black dotted lines (In **C–G**, I, II, and III represent the induction, maintenance, and recovery stages, respectively). (a–g) Statistical charts of EEG frequency band analysis. **(H)** Chart of EEG power spectral density analysis. (Sleep n = 9, Dex n = 10, Sevo n = 10, Iso n = 10, Pro n = 10, Ket n = 10, Eto n = 10). Paired t-tests were used to calculate all *p*-values. *P ≤ 0.05, **P ≤ 0.01, ***P ≤ 0.001.

**FIGURE 2:**
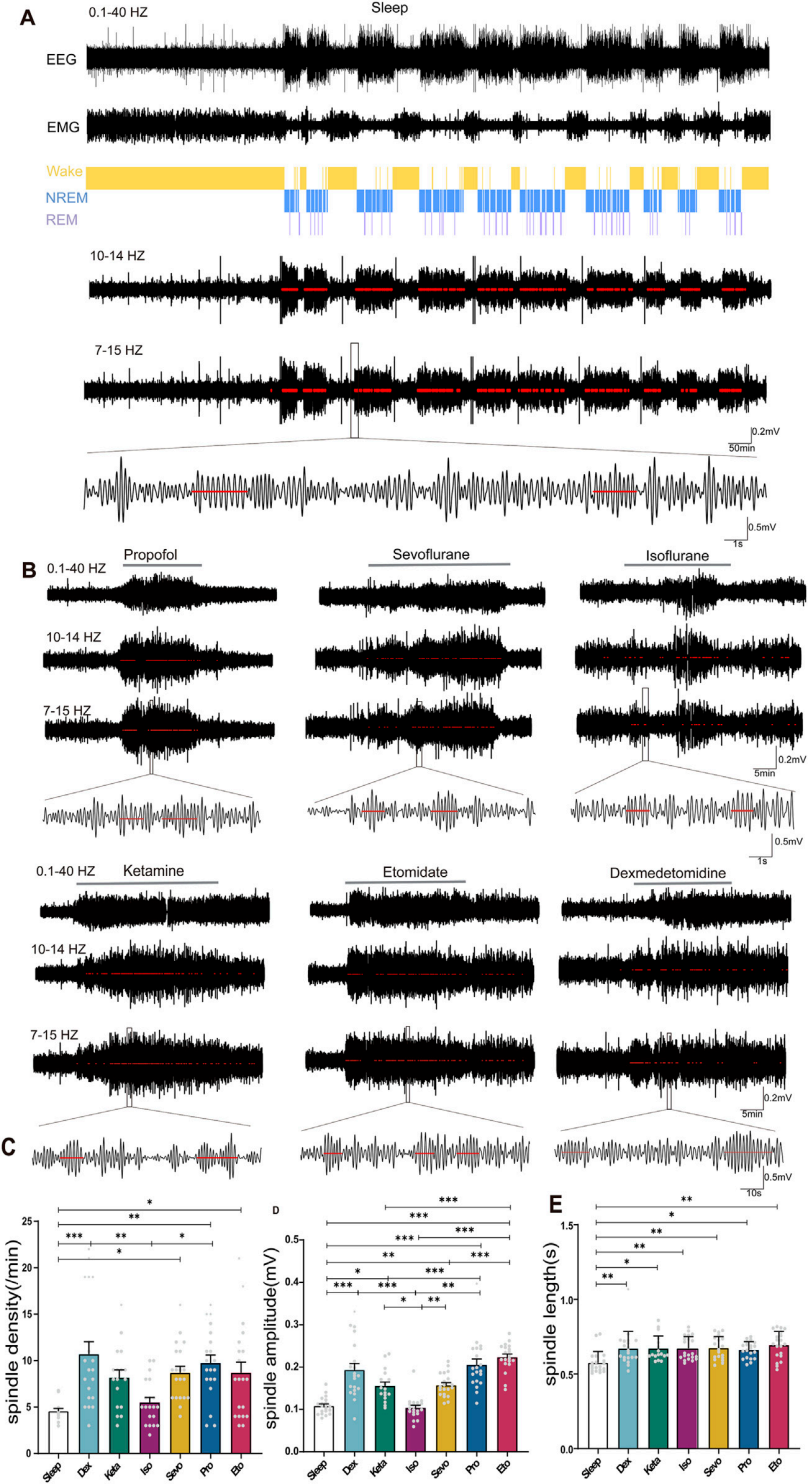
Distinction between sleep spindles and spindles induced by different drugs. **(A)** 24-h electroencephalography (EEG) and electromyogram (EMG) recordings in mice during sleep, with differentiation between rapid eye movement (REM) sleep and non-REM sleep stages. Spindles were identified by band-pass filtering EEG in the 10–14 Hz and 7–15 Hz range, marked by red lines, and the waveform within the black box was magnified (spindle detection counts: Sleep: 3672). **(B)** Detection and magnification of spindles induced by different drugs (marked by red lines, spindle detection counts: Propofol: 117, Sevoflurane: 96, Isoflurane: 59, Ketamine: 116, Etomidate: 153, Dexmedetomidine: 84). **(C)** Density statistical histogram of sleep spindles and drug-induced spindles. **(D)** Amplitude statistical histogram of sleep spindles and drug-induced spindles. **(E)** Length statistical histogram of sleep spindles and drug-induced spindles. **(C–E)** Sleep n = 14, spindle counts:83; Dex n = 20, spindle counts: 214; Sevo n = 20, spindle counts: 174; Iso n = 20, spindle counts: 110; Pro n = 20, spindle counts: 195; Ket n = 17, spindle counts: 139; Eto n = 20, spindle counts: 174). *P*-values were calculated using one-way analysis of variance. *P ≤ 0.05, **P ≤ 0.01, ***P ≤ 0.001.

**FIGURE 3:**
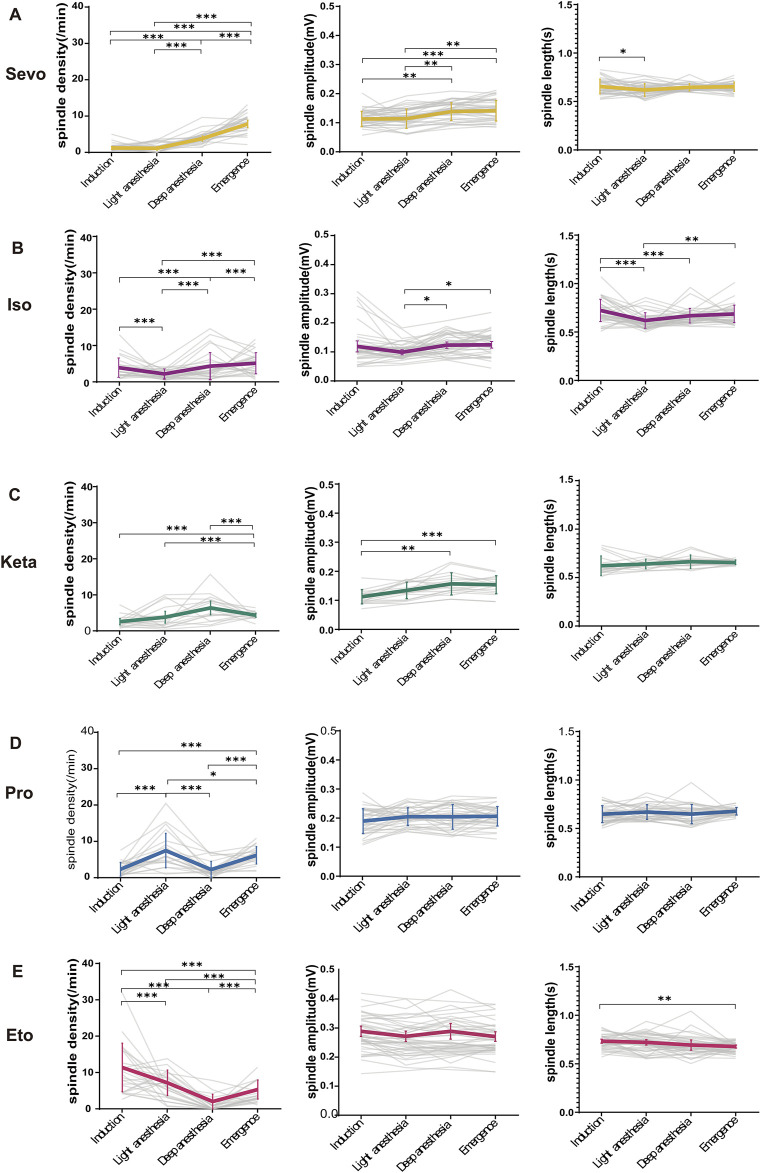
The alterations in anesthetic spindles across four anesthesia stages. **(A–E)** Statistical line charts showing the density (left), amplitude (middle) and length (right) of spindles induced by sevoflurane, isoflurane, ketamine, propofol and etomidate at four anesthesia depths respectively. *P*-values were calculated using one-way ANOVA. (Sevo n = 43, Iso n = 44, Pro n = 39, Ket n = 19, Eto n = 46). *P ≤ 0.05, **P ≤ 0.01, ***P ≤ 0.001.

**FIGURE 4:**
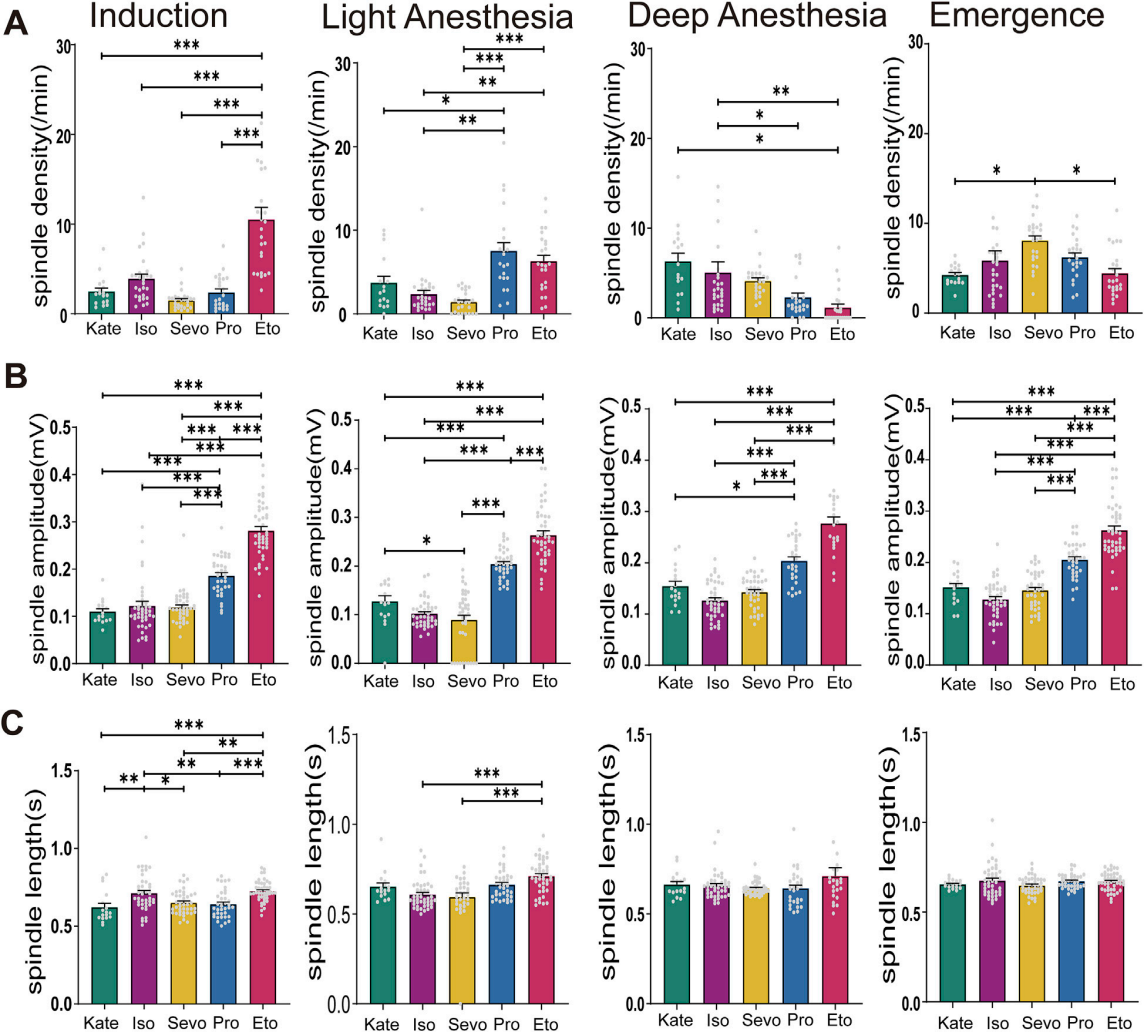
Characteristics of Spindles Induced by Different General Anesthetics at same Anesthesia Stages. **(A)** Density statistical histogram of spindles induced by different general anesthetics at four anesthesia depths. **(B)** Amplitude statistical histogram of spindles induced by different general anesthetics at four anesthesia depths. **(C)** Length statistical histogram of spindles induced by different general anesthetics at four anesthesia depths (Sevo n = 41, Iso n = 42, Pro n = 37, Ket n = 17, Eto n = 44). *P*-values were calculated using one-way ANOVA. *P ≤ 0.05, **P ≤ 0.01, ***P ≤ 0.001.

**FIGURE 5:**
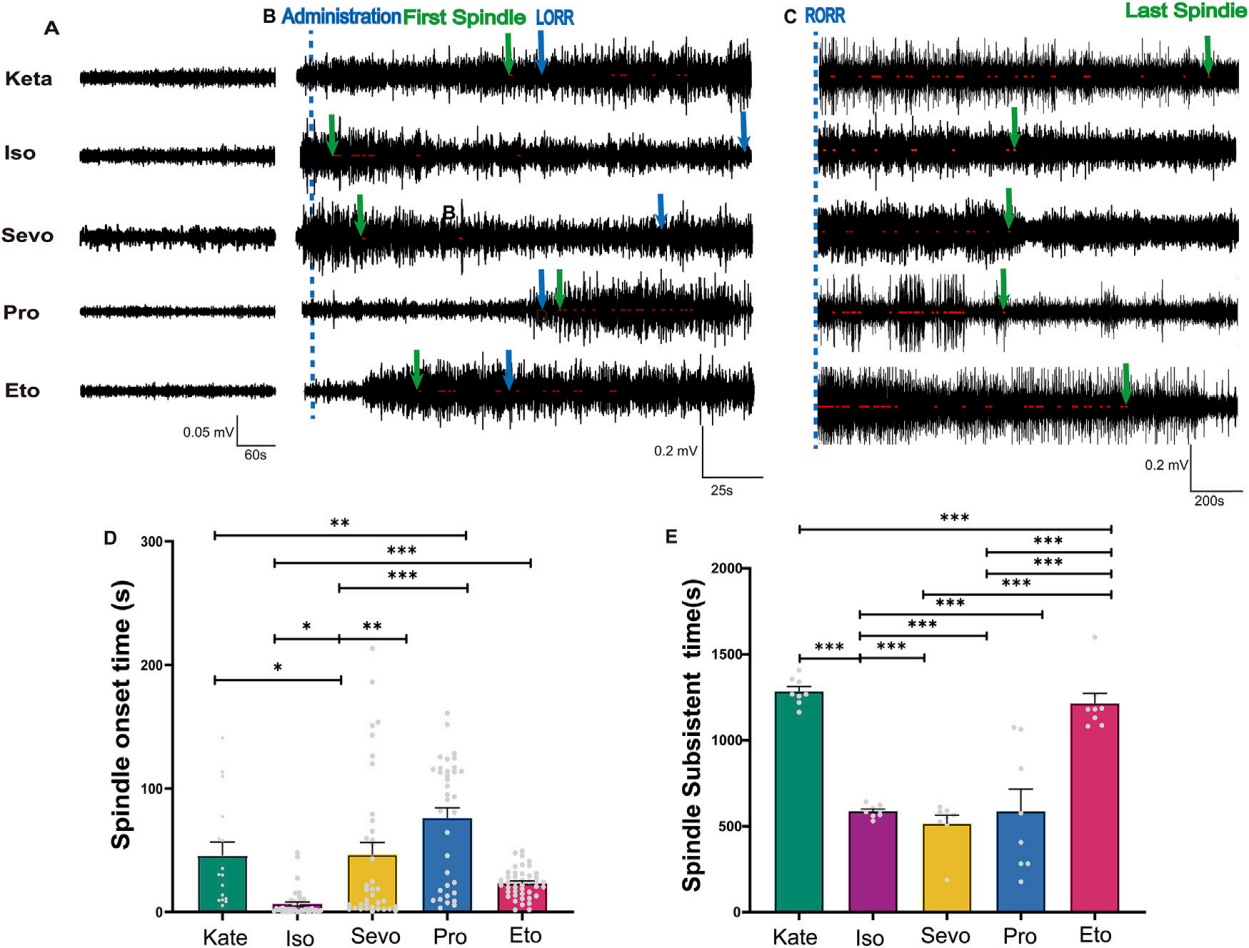
The appearance time and disappearance time of spindles induced by different general anesthetics. **(A)** A sample of EEG traces from a normal waking state (Baseline values before induction = 0.05 mV). **(B)** EEG traces marked with spindles after administration of different general anesthetics, with the administration time indicated by a blue dotted line. The first spindle and loss of righting reflex (LORR) time were marked with green and blue arrows respectively. **(C)** EEG traces marked with post-recovery anesthetic spindles after RORR, with the last spindle indicated by a green arrow. **(D)** Statistical histogram of Spindle onset time (Sevo n = 40, Iso n = 42, Pro n = 37, Ket n = 17, Eto n = 44). **(E)** Statistical histogram of spindle subsistent time (n = 8). *P*-values were calculated using one-way ANOVA. *P ≤ 0.05, **P ≤ 0.01, ***P ≤ 0.001.

**FIGURE 6:**
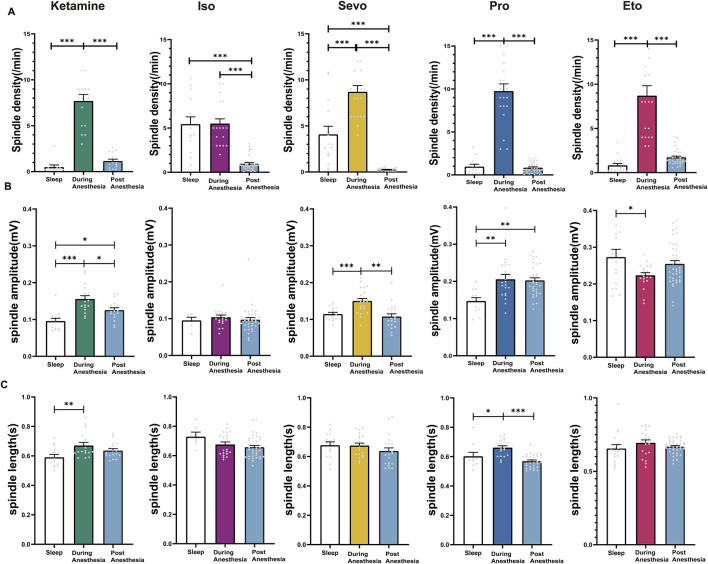
Characteristics of post-recovery Anesthetic Spindles, anesthetic spindles during anesthesia and Sleep Spindles. **(A)** Density statistical histogram of post-recovery anesthetic spindles, anesthetic spindles induced by different general anesthetics and sleep spindles. **(B)** Amplitude statistical histogram of post-recovery anesthetic spindles, anesthetic spindles induced by different general anesthetics and sleep spindles. **(C)** Length statistical histogram of post-recovery anesthetic spindles, anesthetic spindles induced by different general anesthetics and sleep spindles. (Sevo n = 23, Iso n = 37, Pro n = 35, Ket n = 17, Eto n = 40). *P*-values were calculated using one-way ANOVA. *P ≤ 0.05, **P ≤ 0.01, ***P ≤ 0.001.

Each mouse was used for a single EEG recording and was exposed to only one anesthetic agent. The total number of mice reported in Figures 1, 2 reflects the baseline and initial drug testing groups, while the numbers in Figures 3–6 represent the total number of trials across all experiments for each drug condition, which included multiple experimental sessions. No mouse was used in more than one condition.

Criteria for data exclusion included poor-quality EEG recordings, inability to maintain a stable anesthetic state, and technical issues during the recording process. Any mouse that met these exclusion criteria was omitted from further analysis.

This study adheres to the ARRIVE guidelines.

### 2.2 Cortical EEG implantation

Mice were anesthetized with 1.4% isoflurane for induction and maintained at 1% isoflurane, then secured in a stereotaxic apparatus. Cotton coated with vaseline was used to protect their eyes. A 1% lidocaine solution was administered subcutaneously for local anesthesia. The skull was exposed, and stainless-steel screws were implanted in the frontal (anterior-posterior: +1.0 mm; medio-lateral: ±1.5 mm) and occipital (anterior-posterior: −3.5 mm; medio-lateral: ±1.0 mm) regions, ensuring contact with the dura mater. Silver wire electrodes were wrapped around the screws for EEG recording and fixed in place with dental cement. EEG recordings were performed 1 week after the surgical procedure.

### 2.3 Drug administration and anesthetic staging

Different intravenous drugs were administered via tail vein infusion in mice, including dexmedetomidine (Yangtze River Pharmaceutical Group, China, Batch No. 21011331, 10 μg/mL), propofol (Xi’an Libang Pharmaceuticals, China, Batch No. 22103303–1, 10 mg/mL), etomidate (Jiangsu Enhua Pharmaceutical, China, Batch No. YT220501, 2 mg/mL), and ketamine (Fujian Gutian Pharmaceuticals, China, Batch No. 120822, 25 mg/mL). Note that at the beginning of the experiment, the animals were connected to EEG and EMG recording equipment. Following this setup, tail vein puncture was performed, and once successful, the needle was secured to the tail with medical tape. The animal was then placed in the recording chamber, and we recorded 5 min of baseline EEG data during free movement before administering anesthesia. For inhalation anesthesia, the mice were placed in a transparent acrylic induction chamber (25 cm × 14 cm × 17 cm) and exposed to isoflurane (RWD Life Science, China, 1.3%) or sevoflurane (Shandong Bette Pharmaceuticals, China, 2.3%). Specific drug doses and continuous infusion rates are provided in the Supplementary Table S1.

Anesthesia depth was assessed using a combination of physiological indicators, including respiratory rate, the presence or absence of the righting reflex, and the onset of burst suppression (BS) in the EEG. However, BS was not observed in all cases, likely due to specific dosage ranges or the anesthetics used, which may have influenced its appearance. The absence of BS did not necessarily indicate inadequate anesthesia depth, as spindle activity was used as an alternative marker in this study.

Residual volatile anesthetics were removed using an anesthetic gas scavenger (RWD, China). Specifically, anesthetic administration was stopped when burst suppression appeared in the EEG, and the detected anesthetic concentration dropped to 0.4%. At that point, the oxygen flowmeter was set to maximum, and the scavenger was activated to continuously remove any remaining anesthetic gases.

Anesthesia staging in mice was determined by referencing previous studies (Navarro et al., 2021; Jiron et al., 2019) and incorporating several indicators, such as changes in respiratory patterns, the time for loss and recovery of the righting reflex, the onset of BS on the EEG, and other relevant factors. This allowed us to categorize the anesthesia process into distinct stages: induction, light anesthesia, deep anesthesia, and recovery:

Induction stage: Following drug administration, the mice exhibit locomotor activity, increased respiratory rate, unsteady gait, body swaying, and hind limb weakness until the righting reflex is lost.

Light anesthesia stage: The mice lie on their sides, unable to self-correct, show no response to non-painful stimuli, and primarily exhibit thoracic breathing.

Deep anesthesia stage: Respiratory rate decreases, characterized by abdominal breathing and a lack of response to noxious stimuli, with BS eventually appearing in the EEG.

Recovery stage: From the cessation of drug administration until the mice regain their righting reflex.

### 2.4 EEG recording and spindle wave detection

The Apollo portable recording system (Bio-Signal, China) was used to record cortical EEG signals in mice during the four stages of anesthesia, with a sampling frequency of 1,000 Hz. Offline analysis of the raw EEG signals was performed using MATLAB (2016b, The MathWorks Inc., United States) and Spike 2 (Solvusoft Corporation, United States). The initial processing included bandpass filtering (0.1–60 Hz) to remove noise, followed by calculating frequency band ratios within specific ranges (0.5–4 Hz, 4–8 Hz, 8–12 Hz, 12–25 Hz, and 25–60 Hz) from the raw EEG data. Seven days after recovery from surgery, they were placed in the sleep restriction apparatus, and their EEG and EMG recordings were captured by Medusa devices (Biosignal Technologies, Jiangsu, China). Sleep recordings were scored in 4-s epochs over 24 h of normal sleep–wake cycles, sleep restriction, or both. The analysis was conducted semi-automatically through threshold scoring and cluster scoring techniques. NREM EEG epochs were identified as low-frequency delta waves (0.5–4 Hz), characterized by high amplitude (±150–250 μV) with low-amplitude EMG waves. Rapid Eye Movement (REM) EEG epochs were identified by mixed frequencies, primarily in the theta range (6–9 Hz), with low-amplitude EEG waves (±50–100 μV) and flat EMG waves. Wakefulness epochs were scored as high-frequency (8–50 Hz) with low-amplitude EEG waves (±50–100 μV) and high-amplitude EMG waves. Automated scoring of the epochs was visually monitored to ensure accuracy, confirming that each epoch was classified according to the established definitions of wakefulness, NREM, and REM sleep. Any epochs considered to contain movement artifacts were omitted from the data analysis. The sleep stages of the recordings were scored using Lunion Stage, an AI-driven software developed by LunionDate in Shanghai, China (https://www.luniondata.com, Shanghai, China) (Zhai et al., 2023).

The spindle detection method was adapted from previous studies (Niethard et al., 2018). Using custom MATLAB scripts, spindles were detected and quantified from the filtered EEG signals. We chose the band 7–15 Hz based on previous studies that have highlighted its relevance for anesthesia-related spindles (Halassa et al., 2011; Contreras et al., 1997). Additionally, the division into sub-bands (4–8, 8–12, and 12–25 Hz) was implemented to analyze potential differential effects within commonly observed brain oscillatory ranges, which are often subdivided to distinguish specific activity patterns and their functional implications under anesthesia. Specifically, the process involved bandpass filtering the EEG within the 7–15 Hz range, followed by rectification to equalize the positive and negative waveform amplitudes, generating rectified positive waveforms. The goal was to derive the envelope of the EEG signals within this frequency range, depicting the amplitude modulation curve reflecting the signal’s frequency characteristics. A spindle event was identified when the envelope consistently exceeded a predefined threshold for a duration between 0.5 and 3.0 s. This threshold was set at 1.5 times the standard deviation of the entire filtered EEG signal. The onset of the spindle event was marked by the first positive threshold crossing, and the conclusion by the last negative crossing. The root mean square (RMS) value of the identified spindles was averaged for further analysis.

Spindle detection in the EEG traces adhered to stringent criteria to ensure only genuine spindle events were identified. Although some oscillatory patterns in the EEG appeared similar in amplitude and frequency, they were not classified as spindles unless they met specific amplitude and duration thresholds, as defined by previous studies. Spindles were defined as oscillations within the 7–15 Hz range, with a minimum amplitude of 30 nV^2^ and a duration of 0.5–3.0 s. This approach was necessary to avoid misclassifying other types of oscillations that resembled spindles but lacked the physiological characteristics of true spindle events. Consequently, visually similar patterns that did not meet the detection criteria were excluded from the analysis. These thresholds were applied consistently across all EEG recordings to ensure the reliability and reproducibility of spindle detection.

While bandpass filtering within the 7–15 Hz range may reveal oscillatory activity, we ensured the physiological relevance of these oscillations by combining bandpass filtering with validated spindle detection techniques. Applying a bandpass filter to broadband EEG signals can produce oscillations that may not correspond to actual physiological events. To prevent such artifacts, we incorporated additional criteria for spindle detection, including amplitude thresholds and durations consistent with previous literature. This ensured that the detected oscillations were not artifacts of the filtering process but rather reflected true spindle events linked to thalamocortical interactions. Spectral analysis was also performed to further confirm that these oscillations aligned with previously reported spindle characteristics, validating their physiological significance.

### 2.5 Statistical analysis

Statistical analysis and graphical representation were conducted using SPSS (version 26.0, IBM Corp, United States) and GraphPad Prism (version 8.0, GraphPad Software, Inc.). The normality of data distribution was evaluated using the Kolmogorov-Smirnov test. T-tests were employed to compare EEG frequency bands between the awake and post-drug states, as well as to assess variations in anesthetic spindles at different anesthesia depths. Additionally, one-way ANOVA was used to compare the spindle density, amplitude, and duration of anesthetic spindles induced by different anesthetic agents. This approach was also applied to analyze the time to first appearance of anesthetic spindles after drug administration, the time to spindle disappearance after drug withdrawal, and the differences in spindle characteristics across different drugs at equivalent anesthesia depths. Statistical significance was set at *P* < 0.05. Descriptive statistics for continuous variables were presented as mean ± standard deviation (mean ± SD) for clarity.

## 3 Results

### 3.1 Different general anesthetics induced unique EEG spectrograms, frequency band distributions, and power spectral densities

EEG spectrograms, frequency band distribution, and power spectral density are commonly employed methods in EEG analysis for investigating the mechanisms of general anesthesia. Initially, we used these three methods to illustrate EEG patterns during sleep and drug-induced during anesthesia maintenance. As shown in Figure 1, the EEG spectrogram effectively captures variations in EEG activity across different frequencies. For example, during sleep, there are intermittent increases in low-frequency activity (Figure 1A), whereas drug-induced loss of consciousness is characterized by a continuous increase in low-frequency activity. Different anesthetics induced distinct spectrogram patterns (Figures 1B–G).

Frequency band analysis provided a more detailed quantification of changes in EEG energy across different frequency bands (Figures 1A–G). Notably, under etomidate anesthesia, EEG energy in the 1–4 Hz range decreased (*p* = 0.041), whereas for other anesthetics and sleep, EEG energy in this range increased (Sleep: *p* = 0.006, Iso: *p* = 0.0002, Sevo: *p* = 0.0011, Dex: *p* = 0.0041, Pro: *p* = 0.019, Keta: *p* = 0.001241). In the 4–8 Hz range, energy increased during sleep (*p* = 0.007523) but decreased in the other groups (Iso: *p* = 0.001, Pro: *p* = 0.014, Keta: *p* = 0.009). The 8–12 Hz range showed an increase in energy under etomidate (*p* = 0.0008) but a decrease with the other drugs (Sleep: *p* = 0.000073, Iso: *p* = 0.00162607, Sevo: *p* = 0.000626, Dex: *p* = 0.006, Keta: *p* = 0.005817). In the 12–25 Hz range, energy decreased in the sleep and dexmedetomidine groups (Sleep: *p* = 0.0045, Dex: *p* = 0.003) but increased with the other anesthetics (Iso: *p* = 0.040441, Sevo: *p* = 0.000130, Eto: *p* = 0.000585, Pro: *p* = 0.000031). Across the 25–60 Hz frequency band, all groups exhibited decreases in EEG energy (Sleep: *p* = 0.000004, Sevo: *p* = 0.001176, Dex: *p* = 0.000035, Pro: *p* = 0.001, Keta: *p* = 0.000592, Eto: *p* = 0.005091).

Power analysis revealed that both sleep and drug-induced conditions showed peak power within the 2–6 Hz range, indicating similar EEG patterns. The division into 1–4 Hz and 4–8 Hz bands aligns with established spectral analysis methods in both anesthesia and sleep studies, where these divisions help differentiate slow-wave and theta-related activities. The peak observed between 2 and 6 Hz in Figure 1H was thus analyzed within these specific bands to capture distinct oscillatory components relevant to our study focus on neural oscillations under anesthesia. Specifically, dexmedetomidine exhibited a markedly higher peak power spectral density compared to sleep and general anesthesia conditions (Figure 1H). As demonstrated in our results, frequency band distribution and power spectral density primarily reflect overall EEG activity within specific frequency ranges during a given timeframe. However, these methods are limited in capturing continuous dynamic changes. While spectrograms offer insights into energy fluctuations across frequency bands over time, they lack the precision needed to accurately depict the progression of anesthesia from light to deep stages.

### 3.2 Distinctions between anesthetic spindles induced by different general anesthetics and sleep spindles

Using established spindle detection methods (Chen et al., 2023b), we identified and labeled independent spindle events in mice during sleep (Figure 2A) and states of anesthesia induced by dexmedetomidine, three intravenous general anesthetics, and two inhaled general anesthetics (Figure 2B). We specifically analyzed anesthetic spindles occurring within 1 min before the return of righting reflex (RORR) to compare with sleep spindles. The density of anesthetic spindles induced by dexmedetomidine, sevoflurane, propofol, and etomidate were significantly higher than those of sleep spindles (p: Sleep vs. Dex = 0.0004, Sleep vs. Sevo = 0.0487, Sleep vs. Pro = 0.0046, Sleep vs. Eto = 0.0487, F (6, 124) = 5.498, *P* < 0.0001, Figure 2C). The spindle density induced by intravenous anesthetics (propofol) and the sedative (dexmedetomidine) exceeded that of inhaled anesthetics (isoflurane), with no significant difference in spindle density observed between the two inhaled anesthetics (isoflurane and sevoflurane, *p* = 0.1462) or among the intravenous anesthetics (ketamine, propofol, etomidate, p: Keta vs. Pro = 0.8896, Keta vs. Eto = 0.9997, Pro vs. Eto = 0.98) (Figure 2C).

In contrast to sleep spindles, all anesthetic spindles—except those induced by isoflurane—showed higher amplitudes. Etomidate-induced spindles had the highest amplitude (*p* < 0.001), while isoflurane-induced spindles had the lowest (*p* < 0.05, Figure 2D). Spindle amplitudes induced by intravenous anesthetics and the sedative were higher than those induced by inhaled anesthetics (F (6, 130) = 24.13 *P* < 0.0001, Figure 2D). Regarding duration, drug-induced spindles were longer than sleep spindles, with no significant differences in spindle duration across the different drugs (p: Sleep vs. Dex = 0.0085, Sleep vs. Keta = 0.0141, Sleep vs. Iso = 0.0063, Sleep vs. Sevo = 0.0054, Sleep vs. Pro = 0.0263, Sleep vs. Eto = 0.0004, F (6, 131) = 4.163, *P* = 0.0007, Figure 2E). These findings highlight notable differences between anesthetic and sleep spindles, as well as distinct characteristics of spindles induced by different general anesthetics.

### 3.3 Changes in anesthetic spindles across four anesthetic stages

To investigate potential variations in anesthetic spindles across different stages of anesthesia induced by five general anesthetics in mice, we subdivided the unconscious state into four stages: induction, light anesthesia, deep anesthesia, and recovery. As shown in Figure 3A, sevoflurane induced a continuous increase in spindle density, amplitude, and duration throughout the four anesthetic stages (density: *p* < 0.001, F (3, 160) = 88.66, *P* < 0.0001; amplitude: *p* < 0.01, F (3, 150) = 9.116, *P* < 0.0001; duration: *p* = 0.0463, F (3, 150) = 2.810, *P* = 0.0415). In contrast, isoflurane, another inhaled anesthetic, initially suppressed spindle density, amplitude, and duration during the light anesthesia stage, but these parameters increased during the deep anesthesia and recovery stages (density: *p* < 0.001, F (3, 164) = 27.83, *P* < 0.0001; amplitude: *p* < 0.05, F (3, 164) = 3.406 *P* = 0.0191; duration: *p* < 0.05, F (3, 164) = 9.296, *P* < 0.0001; Figure 3B). This suggests that anesthetic spindles could serve as EEG indicators to differentiate the potency of sevoflurane from isoflurane. For ketamine, spindle density and amplitude consistently increased during the light and deep anesthesia stages, followed by a decrease during recovery, while spindle duration remained unchanged across all four stages (density: *p* < 0.001, F (3, 64) = 109.2, *P* < 0.0001; amplitude: *p* < 0.01, F (3, 62) = 7.431, *P* = 0.0002; duration: *p* > 0.05, F (3, 63) = 1.248, *P* = 0.2998; Figure 3C). Propofol-induced spindle density peaked during light anesthesia, significantly decreased during deep anesthesia, and then rose again during recovery (p: Induction vs. Emergence < 0.0001, Light anesthesia vs. Deep anesthesia = 0.0398, Light anesthesia vs. Emergence <0.0001, Deep anesthesia vs. Emergence < 0.0001; F (3, 135) = 106.6, *P* < 0.0001). However, the amplitude and duration of propofol-induced spindles remained consistent across all stages, distinguishing them from those induced by other anesthetics (amplitude: F (3, 135) = 1.624, *P* = 0.1868; duration: F (3, 136) = 1.383, *P* = 0.2506; Figure 3D). Etomidate-induced spindle density decreased as anesthesia deepened, but increased markedly during the recovery phase (F (3, 172) = 85.90, *p* < 0.001). While spindle amplitude remained constant across the four stages (F (3, 151) = 1.201, *P* = 0.3114), spindle duration decreased during recovery compared to the induction stage (p: Induction vs. Emergence = 0.0092, F (3, 151) = 4.065, *P* = 0.0082; Figure 3E).

### 3.4 Different general anesthetics induce distinctive spindles at equivalent stages of anesthesia

Next, we compared the spindle characteristics induced by different general anesthetics at equivalent depths of anesthesia. During the induction stage, spindle density induced by etomidate was notably higher than that induced by other anesthetics (F (4, 114) = 26.4, *P* < 0.0001). In the light anesthesia stage, propofol induced significantly higher spindle density compared to ketamine, isoflurane, and sevoflurane (p: Keta vs. Pro = 0.0023, Iso vs. Pro < 0.0001, Sevo vs. Pro < 0.0001; F (4, 114) = 16.69, *P* < 0.0001). However, in the deep anesthesia stage, spindle density induced by both etomidate and propofol were significantly lower (p: Keta vs. Pro = 0.0088, Keta vs. Eto = 0.0002, Iso vs. Eto = 0.0018, Sevo vs. Eto = 0.0382, F (4, 114) = 6.928, *P* < 0.0001; Figure 4A). Spindle amplitudes induced by etomidate and propofol remained consistently higher across all four anesthesia stages compared to the other three anesthetics (induction: F (4, 176) = 83.95, *P* < 0.0001; light anesthesia stage: F (4, 176) = 101.5, *P* < 0.0001; deep anesthesia stage: F (4, 145) = 61.27, *P* < 0.0001; recovery stages: F (4, 176) = 76.49, *P* < 0.0001; Figure 4B). This suggests a distinct effect of etomidate and propofol on thalamic synchronization, which plays a key role in spindle generation. Spindle duration varied among the five anesthetics only during the induction and light anesthesia stages, with a relatively consistent pattern observed during deep anesthesia and recovery stages (induction: F (4, 176) = 8.586, *P* < 0.0001; light anesthesia stages: F (4, 165) = 9.736, *P* < 0.0001; deep anesthesia stage: F (4, 147) = 1.718, *P* = 0.1490; recovery stages: F (4, 176) = 1.422, *P* = 0.2287; Figure 4C). Overall, spindle density induced by intravenous anesthetics (etomidate and propofol) were higher than those induced by inhalational anesthetics during the induction and light anesthesia stages. However, this pattern reversed during the deep anesthesia and recovery stages.

### 3.5 The appearance and disappearance time of anesthetic spindles induced by different general anesthetics

We defined the duration from drug administration to the initial appearance of anesthetic spindles as the “Spindle Onset Time” (Figure 5A) and the interval from the restoration of the righting reflex to the final disappearance of anesthetic spindles as the “Spindle Subsistence Time” (Figure 5B). Spindles emerged most rapidly following isoflurane administration (6.54 ± 1.67 s), while those induced by propofol appeared the latest (75.95 ± 8.46 s) compared to the other anesthetics (p: Keta vs. Iso = 0.0067, Iso vs. Sevo = 0.0001, Iso vs. Pro < 0.0001, Sevo vs. Pro = 0.0129, Pro vs. Eto < 0.0001; F (4, 167) = 17.20, *P* < 0.0001; Figure 5C). This suggests that the neural substrates contributing to thalamic synchronized activity are highly sensitive to isoflurane and relatively insensitive to propofol. After the restoration of the righting reflex (marking the end of during anesthesia maintenance), anesthetic spindles continued to be observed across all groups, referred to as “post-recovery anesthetic spindles” (p: Keta vs. Iso < 0.0001, Keta vs. Sevo < 0.0001, Keta vs. Pro < 0.0001, Iso vs. Eto < 0.0001, Sevo vs. Eto < 0.0001, Pro vs. Eto < 0.0001; F (4, 35) = 30.82 *P* < 0.0001; Figure 5D). Notably, the spindle subsistence times for ketamine and etomidate exceeded 20 min, whereas for the other three anesthetics, the times were approximately 8 min. Therefore, anesthesia-induced spindles not only promptly and objectively reflect the onset of anesthetic effects but also indicate the residual effects of different anesthetics.

### 3.6 Differences between post-recovery anesthetic spindles, anesthetic spindles during anesthesia and sleep spindles

We compared post-recovery anesthetic spindles, anesthetic spindles during anesthesia, and sleep spindles in the same mice. The results showed that the spindle density of post-recovery anesthetic spindles following inhalation anesthesia (isoflurane and sevoflurane) was significantly lower than that of both sleep spindles and anesthetic spindles (isoflurane: F (2, 68) = 47.60, *P* < 0.0001; sevoflurane: F (2, 55) = 61.15, *P* < 0.0001). In contrast, the spindle density of post-recovery anesthetic spindles after intravenous anesthetics (ketamine, propofol, and etomidate) showed no significant difference compared to sleep spindles (ketamine: F (2, 40) = 68.25, *P* < 0.0001; propofol: F (2, 64) = 119.6, *P* < 0.0001; etomidate: F (2, 72) = 53.19, *P* < 0.0001; Figure 6A). The amplitude of post-recovery anesthetic spindles during ketamine and propofol anesthesia was higher than that of sleep spindles (p: ketamine: Sleep vs. Post = 0.0337, F (2, 44) = 13.69, *P* < 0.0001. propofol: Sleep vs. Post = 0.0017; F (2, 64) = 7.491, *P* = 0.0012), while the amplitude of anesthetic spindles during ketamine and sevoflurane anesthesia was higher than that of post-recovery anesthetic spindles (p: ketamine: During vs. Post = 0.0195; sevoflurane: During vs. Post < 0.0001, Figure 6B). The duration of post-recovery anesthetic spindles across all five anesthetics showed no significant difference compared to sleep spindles (*p* > 0.05, Figure 6C). These findings indicate that post-recovery anesthetic spindles differ from both anesthetic spindles during anesthesia and sleep spindles, confirming that the “drowsy” state observed in mice post-anesthesia is distinct from physiological sleep.

## 4 Discussion

Using a commonly employed method for sleep spindle detection, this study observed spindles during the period of unconsciousness induced by one sedative-hypnotic drug and five general anesthetics. The spindles induced by these anesthetics exhibited distinct variations across different stages of anesthesia, suggesting that anesthetic spindles may serve as unique EEG markers reflecting the varying depths of anesthesia induced by different agents. Notably, the appearance of anesthetic spindles can signify the onset of anesthesia, providing an objective and accurate index for studies focusing on the early stages of anesthesia.

We acknowledge the variability observed in the control condition, particularly in the alpha-gamma range power across different drugs. This variability may be influenced by the individual condition of each mouse prior to the experiment, including factors such as baseline activity levels and sensitivity to anesthesia. To address this, we accounted for inter-subject variability by standardizing experimental conditions and using statistical analyses to reduce the impact of baseline differences. Despite this variability, our results remain interpretable, as we focused on relative changes induced by each anesthetic, rather than absolute baseline values. By analyzing the trends within each drug group and comparing them to the corresponding control, we ensured that our findings reflect the specific effects of each drug on spindle activity. Specific EEG frequency bands.

Furthermore, we observed that post-recovery anesthetic spindles persisted for a prolonged duration, distinguishing them from sleep spindles. This suggests that post-recovery anesthetic spindles could serve as EEG markers indicating short-term sequelae following general anesthesia. The cessation of these spindles may signal the complete recovery of brain function after anesthesia.

In this study, we applied a spindle detection threshold for events lasting between 0.5 and 3 s, in line with previous research on sleep spindles. However, it is important to acknowledge that under anesthesia, spindle-like activity could potentially become continuous. The choice of a maximum duration of 3 s was intended to distinguish transient spindle events from sustained oscillatory patterns that may arise due to the effects of anesthesia. Nevertheless, it is possible that certain spindle-like events, particularly under deep anesthesia, extend beyond this window. Future studies may benefit from exploring whether longer spindle-like oscillations under anesthesia share the same physiological significance as shorter spindles observed during sleep. This could provide a more comprehensive understanding of spindle dynamics and their role in assessing anesthesia depth.

In our study, drug administration was individualized based on the body weight of each mouse. As a result, the actual dosage varied accordingly with each animal. We administered the anesthetics until BS appeared in the EEG, indicating the transition to deep anesthesia. This approach ensured that each mouse progressed through the full stages of induction, light anesthesia, deep anesthesia, and recovery, allowing for a comprehensive assessment of anesthesia depth. By individualizing drug administration, we were able to accurately evaluate the effects of different anesthetics on spindle activity under controlled conditions.

In studies investigating the mechanisms of loss of consciousness during general anesthesia in mice, a widely used indicator is the time to the loss and subsequent recovery of the righting reflex (Wasilczuk et al., 2018). However, the righting reflex only provides information about the initiation and termination of anesthesia, reducing the complexity of general anesthesia to a binary state. This limitation restricts the ability to study neural activity at specific time points that correspond to changes in anesthesia depth.

In this study, anesthesia depth was assessed using a modified version of Guedel’s stages of anesthesia, which primarily focuses on observable parameters such as respiration and movement. However, these parameters can be influenced by the brainstem and spinal cord effects of drugs, which may vary between different classes of anesthetics. To address the variability in anesthesia depth assessment based solely on respiratory movements, we introduced BS in the EEG as an additional indicator to improve the accuracy of depth assessment. The appearance of BS marks the transition into the deep anesthesia stage and has been widely utilized in studies investigating the mechanisms of general anesthesia (Brown et al., 2010). The dosage and effects of anesthetic drugs can be assessed by observing the appearance and duration of burst suppression during anesthesia. Additionally, BS is associated with a poor clinical prognosis. For instance, a prospective study by Momeni et al. identified BS as a risk factor for postoperative delirium (Momeni et al., 2019). Stacie et al. investigated postoperative cognitive function in 105 elderly patients and found that a shorter duration of EEG BS was linked to increased postoperative cognitive dysfunction (Deiner et al., 2015). Furthermore, a study on cardiac surgery conducted by Pedemonte et al. reported that patients who experienced BS during cardiopulmonary bypass (CPB) were more likely to develop postoperative delirium (Pedemonte et al., 2020). Thus, BS offers a fresh perspective for neuroscience research, enhancing our understanding of the brain’s functions and mechanisms. Spindle activities, driven by thalamo-cortical interactions, offer a complementary perspective on anesthesia depth. We demonstrated that anesthetic spindles objectively reflect the onset of anesthesia and evolve with increasing depth. This enables us to establish specific criteria based on changes in spindle activity to more accurately define the beginning and end of each stage of anesthesia.

Previous research has primarily focused on analyzing changes in overall EEG energy related to slow waves, delta waves, and similar patterns (Yi et al., 2023), without examining spindles as independent EEG events. While BS has been used as an independent marker to describe anesthesia depth, its application is mainly limited to the deep anesthesia stage (Brown et al., 2010). In our study, we identified the appearance of spindles post-anesthetic induction as a key indicator of anesthesia onset. These distinct changes in spindle activity provide valuable insight into variations in anesthesia depth induced by specific general anesthetics, though not universally applicable to all agents. We conducted a comparative analysis between commonly used intravenous and inhalational anesthetics and found significant differences in spindle patterns induced by different anesthetics at equivalent stages of anesthesia depth. These findings highlight the potential of spindles as EEG markers for distinguishing the central effects of different anesthetics.

There is growing evidence that spindles observed during general anesthesia exhibit different characteristics compared to those observed during sleep. While sleep spindles are typically in the sigma frequency range (12–15 Hz), spindles during general anesthesia are dominated by alpha activity (8–12 Hz). Additionally, anesthesia-induced spindles may serve different physiological functions, such as reflecting the modulation of cortical-thalamic circuits by anesthetic agents, which is distinct from the role of spindles in memory consolidation and sleep regulation. Studies have shown that spindle activity during anesthesia is influenced by the depth of anesthesia and the specific agents used, leading to variability in their presentation. These differences highlight the need for a more nuanced understanding of spindles in anesthesia, distinct from their role in sleep.

Several studies have highlighted the alpha-dominant nature of anesthesia-induced spindles (Brown et al., 2010; Vijayan et al., 2013), and how these spindles vary in both frequency and amplitude depending on the depth of anesthesia (Akeju et al., 2014). In contrast, sleep spindles are less influenced by external agents and more associated with internal brain rhythms that support cognitive processes, including memory consolidation (Diekelmann and Born, 2010).

It is well established that alpha activity during anesthesia exhibits a bimodal response, increasing as anesthetic dosage rises until the patient loses consciousness, after which it decreases with further increases in dosage. This phenomenon is particularly prominent in clinical studies of anesthetized patients. However, the novelty of our study lies in its controlled approach using animal models, allowing for precise manipulation of drug dosage and conditions. By using different anesthetics, we are able to explore the alpha activity response in a controlled environment, providing more detailed insights into the dose-response relationship and its underlying mechanisms. This adds a new dimension to the existing body of literature on alpha activity during anesthesia, extending our understanding beyond human clinical studies.

It is important to acknowledge the inter-mouse variability in EEG recordings observed during the study. While all mice displayed spindle activity during anesthesia, the exact characteristics of these spindles, such as frequency and amplitude, varied between individuals. This variability may stem from differences in baseline brain activity, anesthetic sensitivity, or underlying physiological differences between the animals. To address this, we applied statistical analyses that accounted for individual variability, focusing on the relative changes in spindle activity across different stages of anesthesia. Despite this inter-mouse variability, the overall trends in spindle characteristics were consistent across animals, supporting the use of spindles as robust markers of anesthetic depth.

EEG spectrograms (Lendner et al., 2020), frequency band analysis (Guo et al., 2023), and power analysis (Li et al., 2022) are conventional EEG tools used to elucidate the effects of general anesthetics. These indicators effectively demonstrate how anesthetics influence specific EEG frequency bands, revealing the distinctive characteristics of different anesthetic agents. For example, intravenous anesthetics induce more low-frequency activity compared to inhaled anesthetics, with substantial low-frequency EEG activity persisting even after the recovery of consciousness (Figures 1E–G). In contrast, inhaled anesthetics like sevoflurane show a rapid restoration of EEG activity across different frequency bands back to the awake state post-recovery (Figures 1C, D). While bandpass filtering reveals oscillations within this frequency range, it may not fully separate these from overlapping broadband activity, and further studies are needed to validate these observations as discrete physiological signals. Moreover, while EEG frequency band analysis provides valuable insights into overall changes induced by anesthetics over time, it lacks the high temporal resolution needed to accurately interpret EEG alterations across different stages of anesthesia. While bandpass filtering reveals oscillations within this frequency range.

In Figure 1, graph H shows the absolute power spectral density (PSD) across different frequency bands, while graphs b and c represent relative power compared to the control condition. The differences observed, such as the greater power of dexmedetomidine compared to sevoflurane in the 4–8 Hz range in graph H, but not in graphs b and c, arise from this distinction. Absolute PSD measures the total power in each frequency band, while relative power (refers to the proportion of power in a given frequency band relative to the total power across all frequency bands) normalizes these values against the total power, highlighting changes specific to the drug effects. This explains the apparent discrepancies between the figures, as the interpretation of drug effects can vary depending on whether absolute or relative power is analyzed.

Some studies have employed multi-site EEG recording techniques and sophisticated algorithms to assess anesthetic depth, as demonstrated by Zhang Jun (Liang et al., 2023), Botond Roska (Bharioke et al., 2022), and Yongzhi Huang (Huang et al., 2020). These approaches allow for precise quantification of anesthetic depth from multiple perspectives. However, not all laboratories have the resources to conduct such research, given the need for advanced EEG recording equipment, surgical expertise, and specialized data analysis capabilities. In contrast, recording and detecting anesthetic spindles is relatively straightforward, requiring only a single-channel EEG recording device.

Professor Purdon’s research has focused on the effects of multifarious anesthetic agents, including dexmedetomidine (Akeju et al., 2016a), propofol (Weiner et al., 2023), sevoflurane (Cornelissen et al., 2015), and ketamine (Akeju et al., 2016b), on EEG oscillations and brain states in humans. The research demonstrated similarities between EEG patterns observed during dexmedetomidine sedation and propofol-induced anesthesia, with dexmedetomidine-induced spindle oscillations (12–16 Hz) resembling propofol-induced alpha (8–12 Hz) oscillations. Similar to sleep spindles, a characteristic feature of dexmedetomidine-induced loss of consciousness is the maximal power and coherence of spindles occurring at ∼13 Hz, which differ in power spectrum and coherence from propofol-induced alpha oscillations s (Akeju et al., 2014). In our study, we found that compared to sleep spindles, dexmedetomidine- and propofol-induced spindles in mice exhibited significantly higher spindle density, amplitude, and duration.

Alpha oscillations during propofol-induced loss of consciousness are more coherent, with amplitudes approximately 3.9 times greater than those of dexmedetomidine-induced spindle oscillations (Akeju et al., 2014). However, in our study, we found no significant differences in the amplitude, density, or duration of spindles induced by dexmedetomidine and propofol in mice. This discrepancy may be attributed to the smaller and less distinct characteristics of spindles in rodents compared to humans (Miyawaki et al., 2017). Additionally, despite interspecies differences in cortical and thalamic structures, oscillations induced by propofol in the frontal cortex of rodents exhibit similarities to those observed in human scalp EEG (Flores et al., 2017).

In Figure 3, spindle density may not be linearly correlate with depth anesthesia. However, spindle features such as frequency shifts and amplitude changes still provide valuable insights into the anesthetic state, particularly when viewed alongside traditional metrics. It is important to note that although spindle wave activity alone may not fully reflect depth anesthesia, its combination with other physiological indicators can enhance assessment accuracy.

TRN serves as the source of spindle activity (Steriade et al., 1993). The presence of spindles signifies a shift from complex neuronal activity, essential for thalamocortical transmission and cortical encoding, to simpler, synchronized activity, thereby limiting the central integration of input information (Wimmer et al., 2012). Increased spindle density suggests enhanced thalamocortical resonance and correlates with reduced cortical information processing capabilities. Larger spindle amplitudes indicate a greater dominance of burst firing activity by the TRN, further inhibiting thalamic information transmission (Normand et al., 2016). Our findings (Figure 4) reveal that etomidate-induced anesthetic spindles during the induction stage exhibit significantly higher density compared to other general anesthetics, indicating a more pronounced inhibition of cortical information processing. Additionally, the amplitude of etomidate-induced spindles remains elevated across all four anesthesia stages, reflecting robust inhibition of thalamocortical transmission by etomidate. In contrast, the inhaled anesthetic sevoflurane shows persistently high spindle frequencies during the recovery phase, suggesting a sustained inhibitory effect on cortical function. This may explain clinical observations where sevoflurane, compared to propofol, is associated with an increased incidence of postoperative cognitive dysfunction (Geng et al., 2017) and delayed neurocognitive recovery in elderly patients (Zhang et al., 2018). However, further experimental validation is needed to support these hypotheses.

Research on sleep spindle waves has a longstanding history, emphasizing their potential as early predictors of neurological disorders. A decreased density of sleep spindles has been linked to conditions such as schizophrenia (Latreille et al., 2015), while spindle amplitude is associated with ion channel function in TRN neurons. Impaired ion channel function in these neurons may contribute to attention deficits and epileptic disorders (Andrade et al., 2016).

The high variability observed in spindle density in the sleep condition (Figure 6A), particularly before the administration of ketamine and isoflurane, can be attributed to differences in the physiological states of the mice prior to the experiment. Variability in baseline activity or the sleep stages may affect the pre-drug spindle density. Furthermore, anesthetics have different mechanisms of action, which may modulate spindle activity in distinct ways. Isoflurane tends to preserve spindle activity, which explains the higher density observed in this group, while ketamine, with its NMDA receptor antagonism, may disrupt normal spindle generation, leading to the lower spindle density prior to drug administration. This drug-specific modulation likely contributes to the observed differences in spindle density between conditions.

Surprisingly, our findings reveal the persistence of post-recovery anesthetic spindles induced by five different general anesthetics, with ketamine and etomidate exhibiting the longest duration, lasting up to 26 min. In contrast, post-recovery spindles induced by the other anesthetics typically disappeared within 9 min (as shown in Figure 5E). Inhalational anesthetics are known for their rapid pharmacokinetics, resulting in faster recovery from anesthesia compared to intravenous agents. This study demonstrates that spindles, as objective EEG markers, provide a more precise assessment of pharmacokinetic differences between inhalational and intravenous anesthetics. These findings suggest that anesthetic spindles could serve as a novel indicator for investigating changes in cognitive, learning, and emotional states following general anesthesia, including phenomena such as postoperative agitation in children (Fattahi-Saravi et al., 2021) and postoperative delirium (Card et al., 2015). Furthermore, research suggests that both propofol and ketamine may contribute to the development of traumatic memories, potentially leading to long-term changes in cognitive and emotional processes and an increased risk of stress-related disorders (Morena et al., 2017). This may also be linked to the extended duration of post-recovery spindles induced by these two anesthetics.

Postoperative sleep-wake disorders (SWD) frequently occur in patients undergoing surgeries with general anesthesia, manifesting as alterations in sleep structure, subjective sleep quality, or abnormal sleep behaviors (O’gara et al., 2021). The use of general anesthesia during the perioperative period may be an independent risk factor for postoperative SWD (Mashour et al., 2021). As our results demonstrate, spindles induced by inhalational anesthesia generally disappear quickly, while those induced by intravenous anesthetics such as propofol and ketamine take longer to vanish, suggesting a greater impact of intravenous anesthetics on the sleep system compared to inhalational agents. This aligns with research indicating that propofol significantly enhances medial prefrontal cortex (mPFC) functional connectivity during sleep deprivation in animals, more so than the inhalational anesthetic sevoflurane (Zhu et al., 2023). Additionally, ketamine anesthesia has been shown to improve postoperative sleep more effectively than other anesthetics, particularly in elderly individuals and patients with Parkinson’s or Alzheimer’s diseases (Lazic et al., 2015). Our findings suggest that the prolonged presence of post-recovery anesthetic spindles could serve as a valuable EEG indicator for investigating the effects of general anesthesia on sleep, specifically in understanding the role of the thalamic reticular nucleus (TRN) in the regulation of postoperative SWD.

## 5 Conclusion

In summary, our study confirms that the appearance of anesthetic spindles serves as an objective EEG marker, indicating the onset of general anesthesia. Anesthetic spindles also reflect variations in anesthesia depth induced by different agents. These spindles provide a high temporal resolution tool for investigating the mechanisms of consciousness loss during general anesthesia, as well as for exploring changes in emotions, cognition, and sleep states following anesthesia. Although spindles are observed in both general anesthesia and sleep, they represent distinct physiological phenomena. Anesthetic spindles, characterized by prominent alpha activity, reflect the brain’s response to anesthetic modulation, in contrast to the sigma spindles of sleep, which play a role in memory consolidation. Future research should further explore the unique characteristics of anesthesia-induced spindles and their potential for monitoring anesthesia depth.

## Data Availability

The original contributions presented in the study are included in the article/Supplementary Material, further inquiries can be directed to the corresponding author.
