# Supplementary material for: Anesthetic spindles serve as EEG markers of the depth variations in anesthesia induced by multifarious general anesthetics in mouse experiments
Source: Front Pharmacol. 2024 Dec 13;15:1474923. doi: 10.3389/fphar.2024.1474923 (PMC11671261; doi:10.3389/fphar.2024.1474923)
Supplement: Supplementary file 1 [file Table1.docx]

| Drug | dose | volume/time |
| --- | --- | --- |
| Propofol | 15mg/kg | 10mg·kg^‒1^·h^‒1^ |
| Dexmedetomidine | 8μg/kg | 0.65 μg·kg^‒1^·h^‒1^ |
| Etomidate | 10 mg/kg | 15mg·kg^‒1^·h^‒1^ |
| Ketamine | 75mg/kg | 5mg·kg^‒1^·h^‒1^ |
| Isoflurane | 1.3% | 1.3% |
| Sevoflurane | 2.3% | 2.3% |

Table 1 Dose and continuous intravenous infusion rate.

The spectrogram calculation method:

The power spectrum is calculated for the specified number (Number of Shifts) of windows.

For each window:

1. For a timestamped variable, the rate histogram is calculated and copied to the Signal array. The following parameters are used:

Histogram_Start = Start + Shift * (Window_Number - 1)

Bin = 1. / (2. *Maximum_Frequency)

NumberOfBins = 2*Number_of_Frequency_Values

2. For a continuous variable, Window_Start = Start + Shift * (Window_Number - 1)

N values of the continuous variable (starting with the value at Window_Start) are copied to Signal array where N = 2*Number_of_Frequency_Values.

3. Signal values are pre-processed according to the specified Window Preprocessing.

4. Signal values are multiplied by the coefficients of the specified Windowing Function.

5. Discrete FFT of the result is calculated.

6. Power spectrum is calculated from FFT using the formulas defined in (Press et al.,

Numerical Recipes in C., Cambridge University Press, 1992) ^[1]^ ^[2]^

[1] Maisog J M, Chmielowska J.An efficient method for correcting the edge artifact due to smoothing[J].Hum Brain Mapp,1998, 6 (3): 128-36.

[2] Press WH, Teukolsky SA, Vetterling WT, Flannery BP (1992): Numerical Recipes in C: The Art of Scientific Computing, 2nd ed. Cambridge: Cambridge University Press, pp 498, 521-525.
